# Gene regulatory network analysis defines transcriptome landscape with alternative splicing of human umbilical vein endothelial cells during replicative senescence

**DOI:** 10.1186/s12864-021-08185-x

**Published:** 2021-12-02

**Authors:** Momoko Ohori, Yusuke Nakayama, Mari Ogasawara-Shimizu, Hiroyoshi Toyoshiba, Atsushi Nakanishi, Samuel Aparicio, Shinsuke Araki

**Affiliations:** 1 Research, Takeda Pharmaceutical Company Limited, 26-1, Muraoka-Higashi 2-chome, Fujisawa, Kanagawa Japan; 2 Present address: Discovery Technology Research Laboratories, Tsukuba Research Institute, Ono Pharmaceutical Co., Ltd, 17-2 Wadai, 300-4247 Tsukuba, Ibaraki Japan; 3 Present address: Life Science AI, FRONTEO Healthcare Inc., 2-12-23 Konan, Minato-ku, 108-0075 Tokyo, Japan; 4 Molecular Oncology, BC Cancer Agency, 675 W10th Avenue, V5Z 1L3 Vancouver, BC Canada; 5 Department of Pathology and Laboratory Medicine, University of British Columbia, V6T 2B5 Vancouver, BC Canada

**Keywords:** Replicative senescence, HUVEC, Alternative splicing, Microexon, MYC, SIRT2

## Abstract

**Background:**

Endothelial cell senescence is the state of permanent cell cycle arrest and plays a critical role in the pathogenesis of age-related diseases. However, a comprehensive understanding of the gene regulatory network, including genome-wide alternative splicing machinery, involved in endothelial cell senescence is lacking.

**Results:**

We thoroughly described the transcriptome landscape of replicative senescent human umbilical vein endothelial cells. Genes with high connectivity showing a monotonic expression increase or decrease with the culture period were defined as hub genes in the co-expression network. Computational network analysis of these genes led to the identification of canonical and non-canonical senescence pathways, such as E2F and SIRT2 signaling, which were down-regulated in lipid metabolism, and chromosome organization processes pathways. Additionally, we showed that endothelial cell senescence involves alternative splicing. Importantly, the first and last exon types of splicing, as observed in *FLT1* and *ACACA*, were preferentially altered among the alternatively spliced genes during endothelial senescence. We further identified novel microexons in *PRUNE2* and *PSAP*, each containing 9 nt, which were altered within the specific domain during endothelial senescence.

**Conclusions:**

These findings unveil the comprehensive transcriptome pathway and novel signaling regulated by RNA processing, including gene expression and splicing, in replicative endothelial senescence.

**Supplementary Information:**

The online version contains supplementary material available at 10.1186/s12864-021-08185-x.

## Introduction

Cellular senescence is a permanent state of cell cycle arrest caused by the interruption of cell division with limited replicative capacity, also referred to as replicative senescence [1, 2]. Besides cell cycle arrest, senescent cells exhibit morphological changes including the formation of an enlarged body mass and the up-regulation of senescence-associated β-galactosidase (SA-β-Gal) activity. Multi-signaling pathways induce and maintain senescence, which is characterized by DNA damage, oxidative stress, and telomere shortening [2]. All senescence-associated signaling pathways converge at the activation of cyclin-dependent kinase inhibitors p16 (*CDKN2A*), p15 (*CDKN2B*), p21 (*CDKN1A*), and p27 (*CDKN1B*), which are commonly used as molecular markers for senescence [3–5]. Senescent cells accumulate in aged tissues, leading to age-related diseases, such as cancer, cardiovascular disease, diabetes, and neurodegenerative disorders [6, 7]. Endothelial cell senescence is considered a key mechanism of the induction of age-related vascular diseases, such as coronary artery disease, stroke, and hypertension, through vascular endothelial dysfunction [8]. Hence, exploring the signaling pathway and mechanism of endothelial cell senescence is critical for developing disease treatment strategies. The role of oxidative stress and mitochondrial dynamics in endothelial cell senescence has been reported previously [9, 10].

Global transcriptome analysis enables a comprehensive understanding of complex biological processes. Therefore, this technique has been used in several studies to identify senescence-related gene expression changes in inflammatory and mitochondrial pathways in fibroblasts [11–13]. Additionally, alternative splicing (AS) of genes, such as p53, SIRT1, and IGF1, has gained attention as a key determinant of cellular differentiation and aging-associated senescence [14, 15]. However, our current knowledge of the gene regulatory network including genome-wide splicing involved in replicative endothelial cell senescence is limited.

In this study, we conducted a detailed computational transcriptome analysis of senescence-induced human umbilical vein endothelial cells (HUVECs). We identified canonical and non-canonical transcriptional responses induced by cellular senescence in HUVECs compared with those induced by senescence in fibroblasts. Besides gene expression analysis, we clarified the characteristics of AS variants in HUVEC senescence. Our results showed that alternative first exon (AFE) and alternative last exon (ALE) types of splicing were predominantly induced by HUVEC senescence. Further investigation revealed that microexons, which encode small proteins of 1–17 amino acids, are highly evolutionarily conserved and play important roles in various cellular functions and diseases with tissue specificity [16]. Moreover, RNA-seq analysis revealed the alteration of novel microexons during HUVEC senescence. Overall, this study elucidated the transcriptome pathway and novel signaling in HUVEC senescence.

## Results

### Replicative senescence of HUVECs

**Fig. 1:**
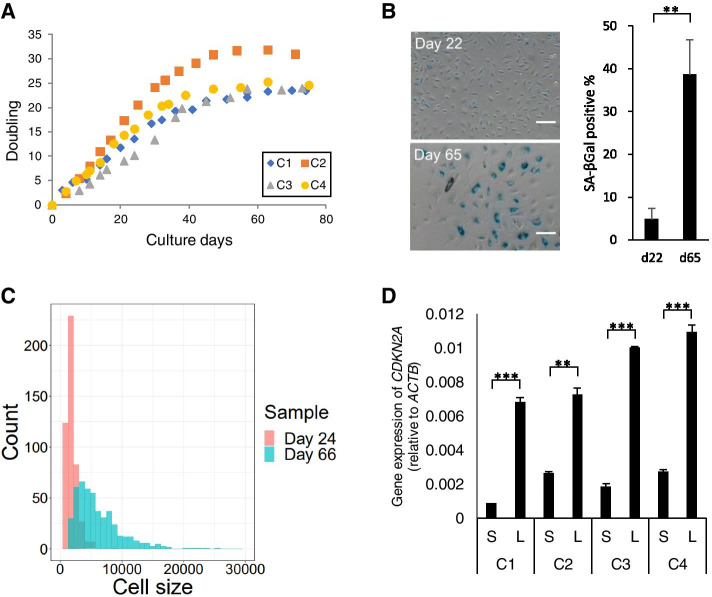
Replicative senescence of HUVEC. (**A**) The doubling rate of HUVECs during replicative senescence. The X-axis indicates culture days. The Y-axis indicates the population doubling (PD) rate. (**B**) Representative pictures and the percentage of SA-β-Gal-stained HUVECs (**P < 0.01; Welch’s t-test). C1 clone of HUVECs cultured for 22 days or 65 days. Scale bar = 100 μm. (**C**) The cellular size of HUVECs during replicative senescence. The Y-axis indicates the number of HUVECs. The X-axis indicates the cell body mass. Pink and blue bars represent HUVECs cultured for 24 and 66 days, respectively. (**D**) The qPCR analysis for the expression of *CDKN2A* of HUVECs during senescence. The Y-axis indicates the relative expression level of *CDKN2A* in four individual clones of HUVECs. The X-axis indicates the time of sampling. S and L indicate short and long culture periods (e.g., day 22 and day 65), respectively. β-actin was used as an internal control. Data represent mean ± standard deviation (SD) of three independent experiments (**P < 0.01; ***P < 0.001; Welch’s t-test)

We prepared senescent HUVECs, which ceased to proliferate, in the presence of a complete culture medium for comprehensive transcriptome analysis. After 60 days of culture, four independent HUVECs (C1–4) stopped dividing, which showed that the population doubling (PD) rate was <0.1 per day (Fig. 1A). Approximately 40–50% of the cells showed SA-β-Gal activity, and the cell size was 3.4-fold greater on day 60 than it was on day 22 (Fig. 1B,C). The expression of *CDKN2A* was induced 10-fold greater on days 73 and 74 than it was on days 5–7 (Fig. 1D). In addition, as shown in Fig. S1, telomere shortening-associated gene, *ISG15*, was up-regulated in a culture period-dependent manner in accordance with fibroblasts and myoblasts with short telomeres [17, 18]. These cellular phenotypes were characteristic of replicative senescent cells, consistent with a previous report [5].

### Comparative transcriptomic analysis of senescent HUVECs and fibroblasts

**Fig. 2:**
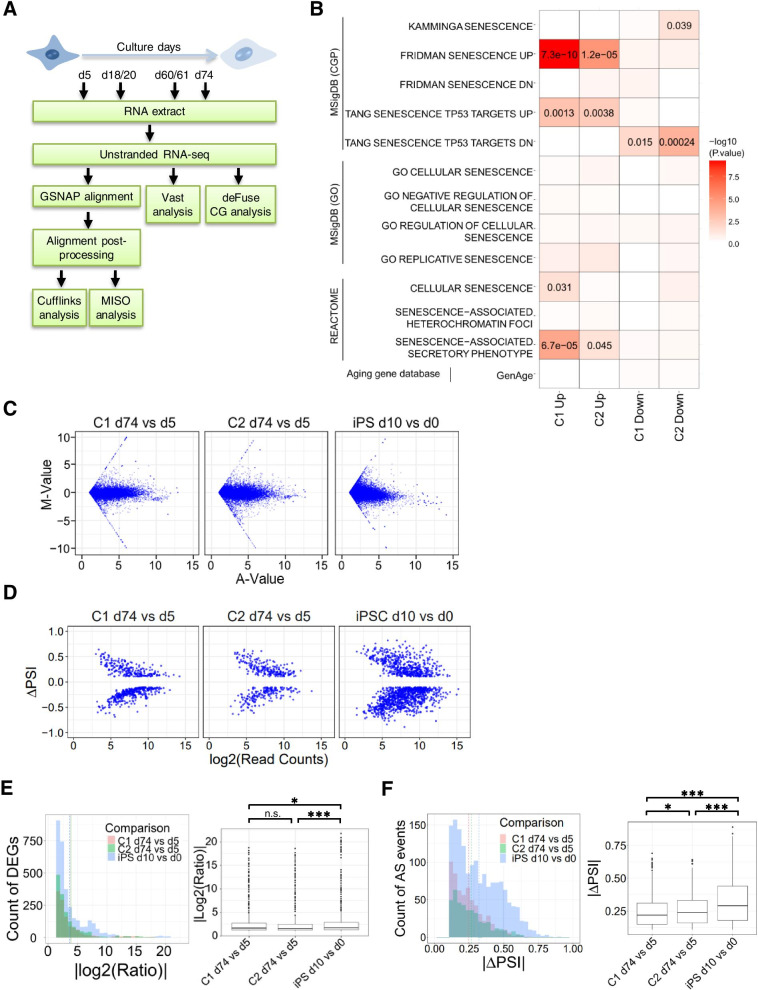
Analysis of DEGs and splicing alteration induced by senescence in HUVECs. (**A**) Flowchart showing the analysis of RNA-seq data. (**B**) Heat map showing the *P*-values (P < 0.05) of enrichment analysis of genes differentially expressed between days 5 and 74 in C1 and C2 clones against known senescence gene sets. (**C**) MA plots of expression profiles of three data sets. The X-axis indicates the FPKM values, and the Y-axis indicates the log2 scale ratio of FPKM in HUVECs of clones C1 (left) and C2 (middle) cultured between 5 and 74 days, respectively, and neuronal differentiation from iPSCs between 0 and 10 days (right). (**D**) Volcano plots of AS events in HUVECs of clones C1 (left) and C2 (middle) cultured for 5 and 74 days and neuronal differentiation from iPSCs for 0 and 10 days (right). The X and Y axes indicate the log2-transformed values of read counts assigned for each splicing event and the ΔPSI value, respectively. (**E**) The distribution of DEGs in C1 and C2 cultured between 5 and 74 days, respectively, and in neuronal differentiation of iPSCs cultured between 0 and 10 days (left). The Y-axis indicates the number of DEGs, and the X-axis indicates the log2 ratio. The dotted line shows the mean log2 ratio. Pink, green, and blue colors represent C1, C2, and iPSCs, respectively: Box plot of the absolute value of the log2 ratio FPKM for HUVECs in clones C1 and C2 cultured between 5 and 74 days, respectively, and in neuronal differentiation of iPSCs cultured between 0 and 10 days (right). The *P*-value was calculated using the Wilcoxon test (*P < 0.05, ***P < 0.001, n.s.: not significant). (**F**) The distribution of AS events in C1 and C2 between 5- and 74-days culture, respectively, and in iPSCs between 0- and 10-days culture (left). The Y-axis indicates the number of AS events, and the X-axis indicates the absolute value of ΔPSI. The dotted line shows the mean value of ΔPSI. Pink, green, and blue colors represent C1, C2, and iPSCs, respectively. Box plot of the absolute value of ΔPSI. Log2 ratio FPKM for HUVECs in clones C1 and C2 between 5- and 74-days culture, respectively, and in neuronal differentiation of iPSCs between 0 and 10 days (right). The *P*-value was calculated using the Wilcoxon test (*P < 0.05, ***P < 0.001)

To investigate transcriptome responses during endothelial cellular senescence, we conducted RNA-seq analysis of two independent HUVEC clones (C1 and C2), with four different culture periods (5, 18/20, 60/61, and 74 days) (Fig. 2A). First, to compare the gene expression signature with the previous data set of senescence-related genes, we conducted hypergeometric test-based enrichment analysis of senescence-related gene sets extracted from three different databases, namely, Molecular Signatures Database (MSigDB), REACTOME, and Human Ageing Genomic Resources (HAGR) [19–21]. Genes showing differential expression between days 5 and 74 (fold change > 2) were significantly enriched among the known senescence-related gene sets (Fig. 2B). The most significantly enriched gene set of “FRIDMAN_SENESCENCE_UP” was previously reported to be up-regulated in senescent cells using the hypergeometric test (C1, *P* < 1 × 10^−9^; C2, *P* < 1 × 10^−4^) [22]. The gene set of the p53-mediated pathway and senescence-associated secretory phenotype was enriched among genes both up- and down-regulated in senescent cells (up-regulated genes: C1 and C2, *P* < 0.005; down-regulated genes: C1, *P* < 0.05; C2, *P* < 0.0005) and up-regulated in senescent C1 and C2 clones (C1, *P* < 1 × 10^−4^; C2, *P* < 0.05). Other senescence-related gene sets did not show significant enrichment with genes showing expression changes during HUVEC senescence. These results suggest that the signature expression pattern of some genes is common to senescent HUVECs and fibroblasts, and the remaining genes are involved in a distinct senescence-related pathway in HUVECs.

Next, we examined the distribution of differentially expressed genes (DEGs) and the alternatively spliced genes (ASGs) between young (day 5) and senescent (day 74) HUVECs. The distributions of fragments per kilobase of exon model per million reads mapped (FPKM) in young and senescent HUVECs are shown in Fig. 2C. ASGs were quantified by MISO analysis, with variation in percent spliced-in (ΔPSI) (Bayes score > 10) as shown in Fig. 2D. As growing body of evidence has indicated the importance of transcriptional regulation and splicing alteration during biological processes, especially those during cellular neuronal differentiation [23, 24], we analyzed DEGs and ASGs during neuronal differentiation from induced pluripotent stem cells (iPSCs) for 10 days [25]. The results showed a three-fold change in FPKM values, thus providing 1222, 1520, and 3227 genes corresponding to C1, C2, and iPSCs, respectively (Fig. 2E). The magnitude of DEGs during senescence was statistically greater than that during neuronal differentiation from iPSCs (mean values: C1, 3.91; C2, 3.80; iPSCs, 3.64 as a scale of log2), as determined by Wilcoxon tests (C1, *P* = 0.04; C2, *P* = 0.0004). Additionally, 637, 451, and 1561 genes were identified in C1, C2, and iPSC data sets, respectively, using the thresholds of ΔPSI > 0.1 and Bayes factor > 10, and the magnitude of ΔPSI in senescent clones was smaller than that in the iPSC data set (mean values: C1, 0.25; C2, 0.27; iPSCs, 0.32), as determined by the Wilcoxon test (C1, *P* < 1 × 10^−22^; C2, *P* < 1 × 10^−9^) (Fig. 2F). These results suggest that the distribution of DEGs was greater than that of the neuronal differentiation of iPSCs and the pattern of ASGs in senescent clones was smaller than that in iPSCs. This motivated us to conduct a more detailed computational analysis of gene expression and AS.

### Key regulators indicating monotonic changes in FPKM during HUVEC senescence

**Fig. 3:**
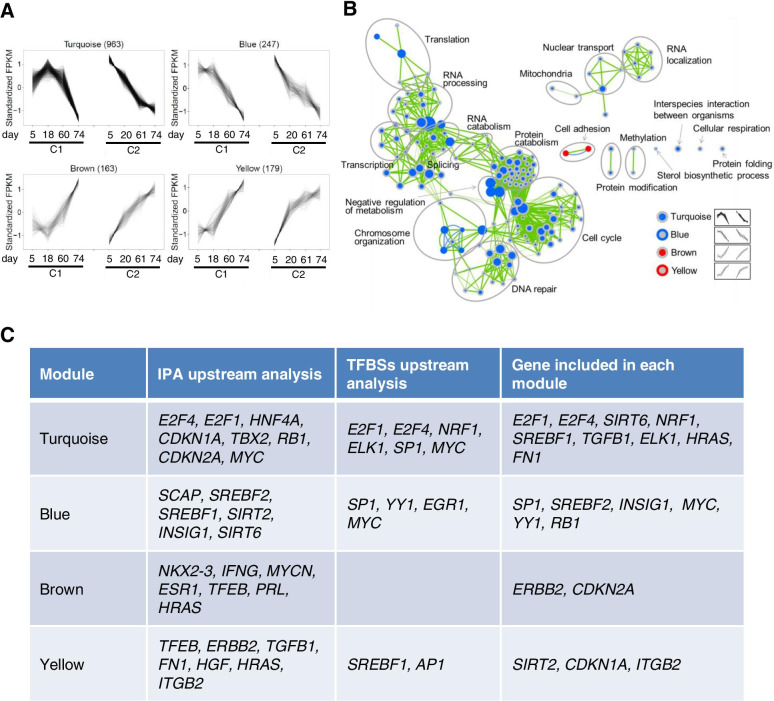
WGCNA of genes showing monotonic decrease or increase in expression (FPKM values) during HUVEC senescence. (**A**) WGCNA of the expression patterns of hub genes in the major co-expressed clusters, based on FPKM values in HUVECs cultured for different durations. The Y-axis indicates the standardized FPKM value. (**B**) Biological process enrichment map of DEGs involved in the culture period-dependent down-regulated (turquoise and blue) and up-regulated (brown and yellow) clusters. Each node represents a GO biological process gene set. The blue circle indicates biological processes enriched among down-regulated genes, and the red circle indicates biological processes enriched among up-regulated genes. Node cores are shown in blue, when the gene set was enriched among the genes, in the turquoise module and are shown in red in the brown module. The outer ring is shown in blue, when the gene set was enriched among genes, in the blue module and is shown in red in the yellow module. Color intensity indicates statistical significance. (**C**) Predicted regulatory network during HUVEC senescence. The first column represents the module name; the second and third columns represent each gene symbol by the IPA upstream analysis and TFBS upstream analysis; the fourth column represents the gene symbol included in each module

To examine the biological significance of responsive transcripts in senescent HUVECs, we conducted Weighted Correlation Network Analysis (WGCNA) of the FPKM transcript data. We identified four dominant FPKM profile modules (turquoise, blue, brown, and yellow) containing 60.6% of the clustered peak events among 20 different modules (Fig. 3A, S2, and S3). These four dominant modules showed a monotonic increase or decrease in responses with the culture period, whereas the other modules showed nonmonotonic patterns in a small number of genes, implying that nonmonotonic patterns detected nondominant effects during senescence (Fig. 3A). Next, we conducted a functional enrichment analysis of each set of four dominant modules to identify the pathways affected by senescence. Only modules with decreasing FPKM (turquoise and blue) showed significant enrichment of biological processes such as cell cycle, DNA repair, metabolism, transcription, translation, splicing, and chromosome organization, as determined using the hypergeometric test (*P* < 1 × 10^−4^) (Fig. 3B, Table S1). Among these, metabolism and chromosome organization processes were down-regulated in both the turquoise and blue modules, implying that key molecules for cellular senescence are included in these processes.

**Fig. 4:**
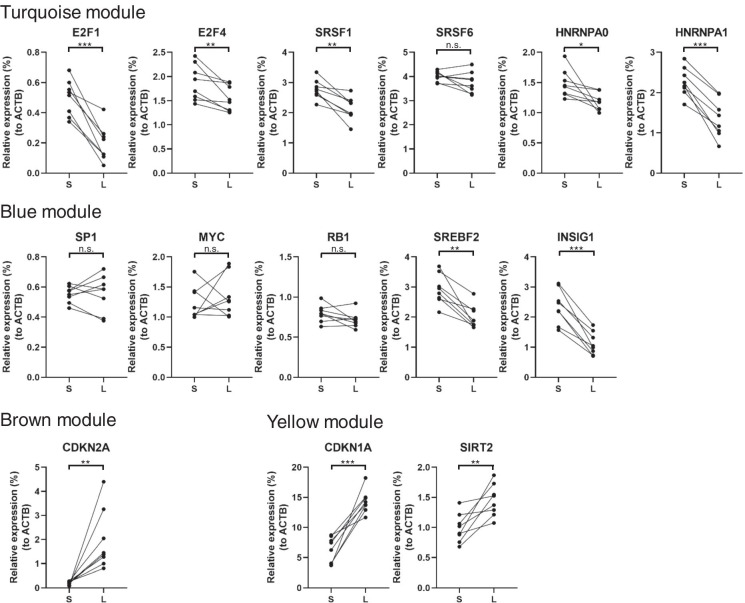
Expression of HUVEC hub genes during senescence. The qPCR analysis of the HUVEC hub gene expression during senescence. The Y-axis indicates the relative expression levels in four individual HUVEC clones (C1–4, N = 2). The X-axis indicates the time of sampling. S and L indicate short and long culture periods (e.g., day 22 and day 65), respectively. β-actin was used as an internal control. The data represents three independent experiments (*P < 0.05, **P < 0.01, ***P < 0.001, n.s.: not significant; Pair’s t-test)

Next, we analyzed the regulatory network of each of the four dominant modules by determining which genes were involved in the regulation of genes in each module. Genes with high connectivity (>0.8) were defined as hub genes; these genes were considered as key genes in a co-expression network [26]. A hub gene has the potential to affect many genes in its network. We identified 963, 247, 163, and 179 hub genes in the turquoise, blue, brown, and yellow modules, respectively (Fig. S4). To predict the transcriptional regulatory network of hub genes, we conducted a computational analysis using two different databases: the ingenuity pathway analysis (IPA) upstream database and transcription factor binding site (TFBS) database [27, 28]. The IPA upstream regulator analysis revealed potential upstream regulators of genes in each module, and upstream gene analysis using TFBSs in MSigDB revealed upstream gene candidates for each module (Fig. 3C). Canonical senescence-related molecules, identified as upstream regulators such as *E2F1*, *E2F4*, *RB1*, *CDKN1A*, *CDKN2A*, and *MYC*, were included in the turquoise module. Interestingly, the sirtuin (SIRT) family, *SIRT2* and *SIRT6*, was identified as an up-regulator of genes in the blue module. *SIRT2* was included in the yellow module as a gene showing a monotonic increase in expression. SIRT1 is a well-known senescence-related SIRT family protein; however, the role of SIRT2 in senescence remains unclear. In addition, the lipid metabolism-related family (*SREBF1*, *SREBF2*, *INSIG1*, and *SCAP*) was included as an up-regulator in the blue module (Fig. 3C). Next, we examined the hub gene expression levels using 4 independent senescence-induced HUVEC clones (C1–4; n = 2 each). Although the gene expression of *MYC*, *SP1*, and *RB1* did not significantly change during HUVEC senescence, other canonical senescence-related molecules, *E2F1*, *E2F4*, *CDKN1A*, and *CDKN2A*, were significantly altered during senescence (Fig. 4). The expression of the non-canonical hub genes *SREBF2*, *INSIG1*, and *SIRT2* in the blue and yellow modules significantly changed during senescence.

Next, to examine whether the identified hub genes were involved in the recent single-cell senescence analysis, we validated their expression levels in each cluster (clusters 0 and 1: quiescent cells; clusters 2 and 3: up-regulation of senescence-associated secretory phenotype-related genes) defined by the previous analysis [29]. The expression of several hub genes, including *HNRNPA0*, *HNRNPA1*, and *CDKN1A*, were also significantly altered in HCA2 fibroblasts with replicative senescence as determined using the Wilcoxon tests (*P* = 1.3 × 10^−9^, *P* = 5.4 × 10^−27^, *P* = 5.5 × 10^−37^, respectively), though the remaining hub genes were not detected in the single-cell analysis due to the lower detection sensitivity of the sequencing platform (Fig. S5). These results suggest that several senescence-related hub genes are consistent between HUVECs and fibroblasts with replicative senescence. Taken together, WGCNA and upstream pathway analysis suggest that canonical and non-canonical senescent pathways, such as E2F and sirtuin signaling pathways, regulate HUVEC senescence.

### Predominant changes in AFE- and ALE-type splicing during HUVEC senescence

**Fig. 5:**
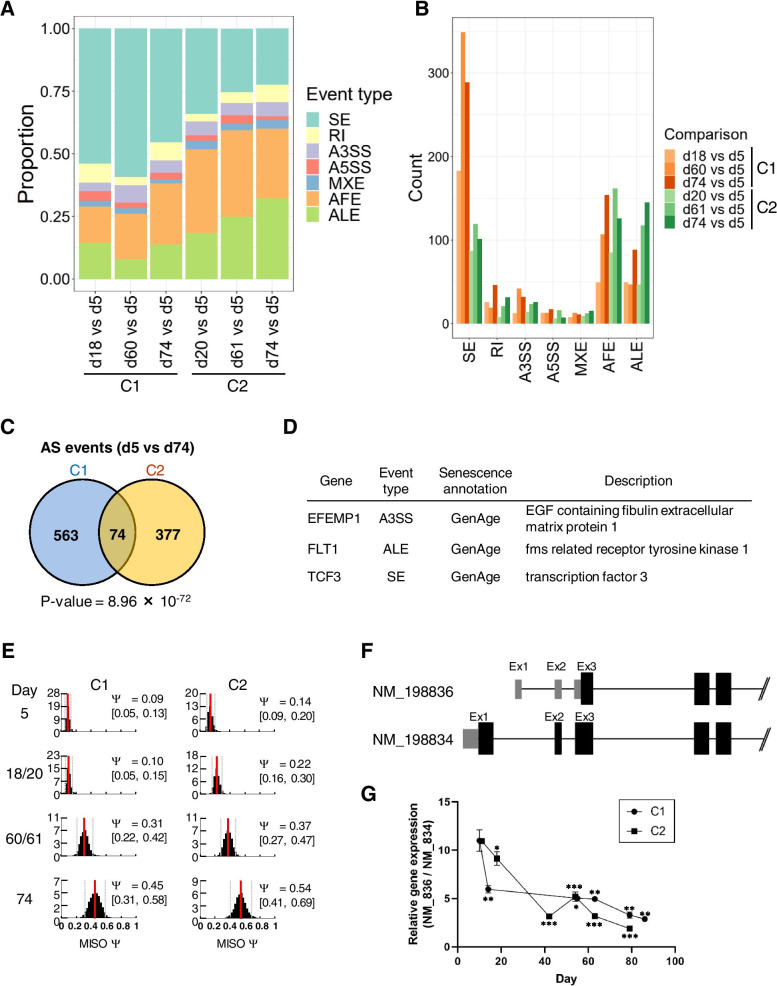
Predominant changes in AFE- and ALE-type splicing during HUVEC senescence. (**A**) Proportions of different types of splicing events in HUVECs in each culture period, as determined by MISO analysis. (**B**) Splicing event type counts for each comparison. (**C**) Venn diagram showing the overlap between AS events between day 5 and day 74 in C1 and C2 clones. Statistical significance was determined using Fisher’s exact test. (**D**) List of alternatively spiced genes related to senescence. (**E**) The ΔPSI distribution for the splicing of *ACACA* in C1 and C2 clones during senescence. The Y-axis indicates the FPKM values, and the X-axis indicates the MISO PSI posterior distribution in both C1 (left) and C2 (right) at the indicated culture condition. (**F**) Schematic representation of two spliced isoforms, NM_198836 and NM_198834, of *ACACA* mRNA. Light-colored boxes represent untranslated exons, and dark-colored boxes represent translated exons. Lines represent introns. (**G**) Analysis of *ACACA* gene expression by qPCR. The Y-axis indicates relative gene expression (NM_198836 [NM_836]/NM_198834 [NM_834]). The X-axis indicates culture days. Data represent mean ± SD of two independent experiments (*P < 0.05, **P < 0.01, ***P < 0.001; Welch’s t-test)

To examine how HUVEC senescence affects AS, we quantitated the AS events between senescent HUVECs at early (day 5) and late (days 60–74) timepoints during culture (Fig. 5A). We classified the splicing events as alternative 3ʹ splice sites (A3SSs), alternative 5ʹ splice sites (A5SSs), AFEs, ALEs, mutually exclusive exons (MXEs), retained introns (RIs), and skipped exons (SEs) (Fig. 5 A,B). The proportion of AFE- and ALE-type splicing increased after HUVEC senescence, and the fold increase in the number of AFEs and ALEs was greater than that in the number of SEs in both C1 and C2 clones during senescence (AFE: 3.1- and 1.5-fold; ALE: 1.8- and 3.1-fold; SE: 1.6- and 1.2-fold, respectively). The ΔPSI magnitude was not significantly different among the splicing events (Fig. S6). Among the alternatively spliced events between day 5 and day 74 in HUVEC culture, 74 splicing events overlapped between C1 and C2 clones in a statistically significant manner, as determined by Fisher’s exact test (*P* = 8.96 × 10^−72^), suggesting that the identified ASGs depend on HUVEC senescence, not reflecting the difference between C1 and C2 (Fig. 5C). Some of the overlapped splicing events were detected in three senescence-related genes (*EFEMP1*, *FLT1*, and *TCF3*) (Fig. 5D, Table S2). A3SS-, ALE-, and SE-type splicing was altered during senescence in *EFEMP1*, *FLT1*, and *TCF3*, respectively. Among the ASGs, except senescence-related genes, the ΔPSI distribution for the AFE-type splicing of *ACACA* (acetyl-CoA carboxylase-α [ACCα]) was significantly altered in both C1 and C2 clones during senescence (Bayes factor > 10^12^) (Fig. 5E). Multiple promoters regulate ACCα mRNA, registered as NM_198836 and NM_198834, in the presence of thyroid hormone, glucagon, and medium-chain fatty acids [30] (Fig. 5F). The quantitative PCR analysis revealed that the expression of NM_198836 significantly decreased compared with that of NM_198834 in a time-dependent manner during senescence, implying that AFE-type splicing of ACCα mRNA is involved in HUVEC senescence (Fig. 5G and S7). Taken together, splicing alterations in senescence- and non-senescence-related genes were induced during HUVEC senescence, and AFE- and ALE-type splicing was predominantly altered compared with other types of splicing.

### Microexon alterations during HUVEC senescence

**Fig. 6:**
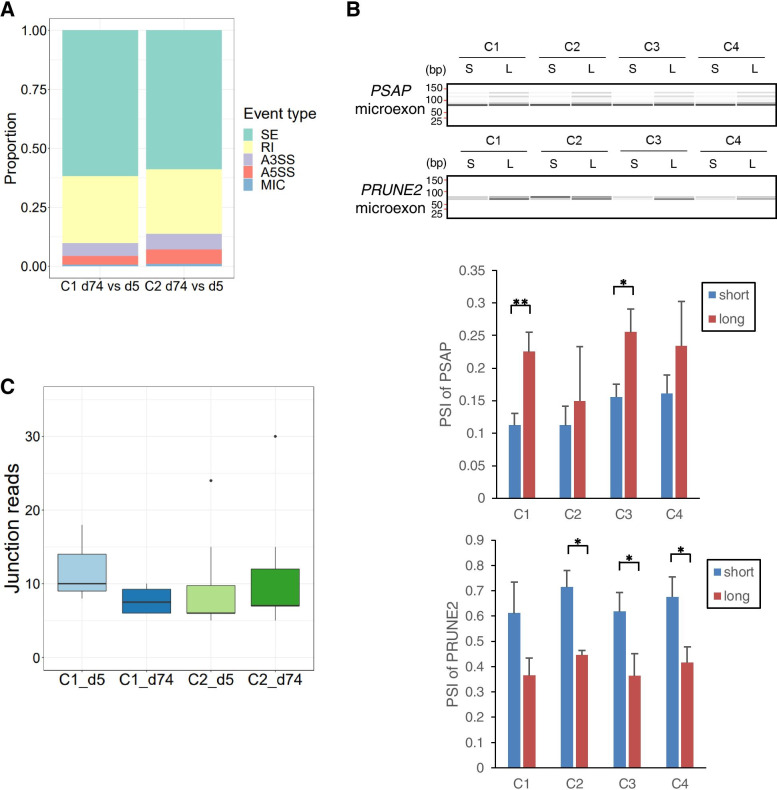
Microexon alterations during HUVECs senescence. (**A**) Proportions of different types of splicing events in HUVECs in each culture period, as determined by VAST-TOOLS. (**B**) RT-PCR assays monitoring AS patterns of microexons in *PSAP* (top) and *PRUNE2* (bottom) in HUVECs under short and long culture periods. PCR products were analyzed using capillary electrophoresis and the intensity of the obtained bands was quantified. PSI values are shown in the bar plot. (**C**) Box plots showing the distribution of conjoined gene junction reads per condition. Data represent mean ± SD of three independent experiments (* *P* < 0.05, ** *P* < 0.01; Welch’s t-test)

Besides canonical splicing analysis, we examined whether microexons are induced by HUVEC senescence using VAST-TOOLS, which detects splicing alterations at all hypothetical splice junctions formed by the usage of annotated and unannotated splice sites [31]. The results of VAST-TOOLS analysis were similar to those of MISO analysis; however, more RI events were detected because of the increased sensitivity for unannotated splice sites (Fig. 6A). We identified several microexon alterations during HUVEC senescence, although to a lesser extent (Table S3). Among these, two microexons in *PRUNE2* and *PSAP*, each containing 9 nt, were altered in both C1 and C2 clones during senescence. To confirm these results, we amplified the microexon-containing region from each isoform by reverse transcription PCR (RT-PCR). Besides C1 and C2 clones, we examined clones C3 and C4. Consistent with VAST-TOOLS results, microexons in *PRUNE2* decreased and microexons in *PSAP* increased the PSI value, with statistical significance or tendency, during HUVEC senescence in all clones (Fig. 6B). Each altered microexon contained the CRAL-TRIO lipid-binding domain and saposin B-type domain in *PRUNE2* and *PSAP*, respectively, implying that microexon alteration affects the function of the encoded protein.

Furthermore, to examine whether readthrough transcripts were induced by HUVEC senescence, we conducted an RNA-seq analysis using deFuse [32]. The readthrough transcript refers to a conjoined gene arising from the upstream transcript to the downstream transcript of partner genes with poly(A) sites [33, 34]. However, RNA-seq data showed that the number of conjoined genes did not change, indicating that HUVEC senescence does not involve conjoined genes (Fig. 6C). Together, our results suggest that a limited number of microexon splicing alterations are induced during HUVEC senescence.

## Discussion

**Fig. 7:**
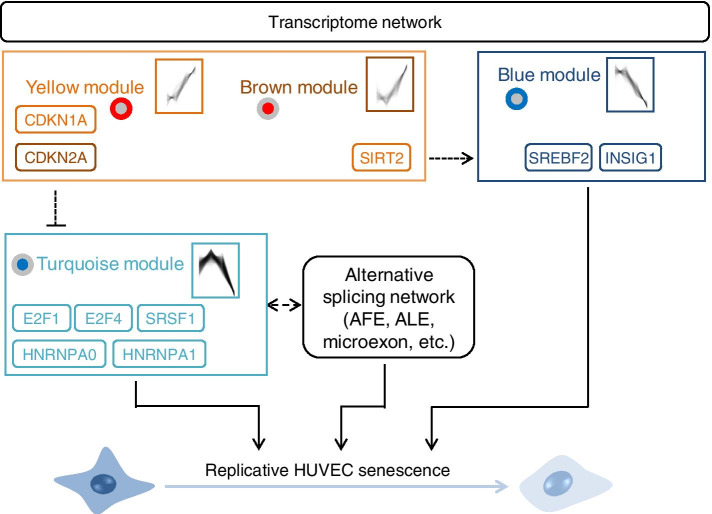
Predicted regulatory network that controls HUVEC senescence

To clarify the transcriptome landscape of HUVECs during replicative senescence, we analyzed changes in gene expression and genome-wide splicing using comprehensive multi-technical RNA-seq analysis. Genes with high connectivity showing a monotonic expression increase or decrease were identified as hub genes. Additionally, canonical and non-canonical senescence pathways were identified during HUVEC senescence. Canonical senescence-related molecules, including E2F family, *CDKN1A* (p21), and *CDKN2A* (p16), were identified from the turquoise module as upstream regulators and confirmed using qPCR analysis. Generally, multiple stress signals, such as DNA damage, inhibit two inhibitory regulators, *CDKN1A* (yellow module) and *CDKN2A* (brown module), and subsequently activate the E2F pathways of the turquoise module (Fig. 7). Total E2F reduction induces senescence-like cell cycle arrest in cancer cells [35]; particularly, E2F1 blocks cell proliferation [36]. Given that chromatin assembly is one of the hub functions of down-regulated biological functions (Fig. 3B), E2F pathways could play key roles in the down-regulation of genes related to cell cycle, transcription, splicing, and translation during HUVEC senescence.

Besides the canonical pathway during senescence, *SIRT2* was identified as one of the genes with monotonically increasing expression in the yellow module and concomitantly identified as an up-regulator in the blue module. The SIRT family proteins function as NAD^+^-dependent histone deacetylases and master regulators of metabolism and aging [37]. *SIRT1* and *SIRT6* are well-known to regulate HUVEC senescence via the regulation of transcription factors, p53, E2F1, and nuclear factor-kappa B [38, 39]. However, the role of SIRT2 in senescence has not been well elucidated, although it is known that SIRT2 is involved in the stress-induced premature senescence, but not in the quiescence, of U2OS osteosarcoma cell line [40]. In addition to *SIRT2*, *SREBF2* and *INSIG1* were also identified as hub genes with monotonically decreasing expression in the blue module. The SREBF protein (SREBP) is involved in cholesterol homeostasis by targeting cholesterol biosynthesis-related genes, such as HMG-CoA reductase [41, 42]. SREBPs are localized in the endoplasmic reticulum membrane binding the SREBP cleavage-activating protein (SCAP) and INSIGs before activation. Since the SIRT family is reportedly involved in cholesterol homeostasis by regulating SREBPs and other factors, the time-dependent up-regulation of *SIRT2* during HUVEC senescence suggests the involvement of a novel SIRT2-related signaling pathway, including lipid metabolism during HUVEC senescence. However, further investigation is needed to confirm whether the identified hub genes, such as E2F, SIRT2, SREBF2, and INSIG1, trigger HUVEC senescence.

Novel splicing alterations during HUVEC senescence were also investigated in this study, although the distribution of ASGs in senescent clones was smaller than the neuronal differentiation of iPSCs. Particularly, AFE- and ALE-type splicing was predominantly induced by senescence. Interestingly, hub genes identified during HUVEC senescence included key splicing factors, such as *SRSF1*, *HNRNPA0*, and *HNRNPA1*, among others (Table S1). Two independent large-scale microarray-based expression cohorts in human blood have revealed that the expression of key splicing factors, SRSFs and hnRNPs, varies with age [43]. Additionally, changes in the expression of splicing factor genes is correlated with cognitive decline and longevity in humans and mice [44]. Presumably, gene expression changes of splicing factors during senescence are involved in splicing alteration and translation, thereby contributing to aging and longevity. We further identified that novel microexon alterations are involved in HUVEC senescence, albeit to a lesser extent. These evidence raise the possibility that induced splicing isoforms preform distinct and cooperative functions during senescence and impact senescence signaling.

## Conclusions

Transcriptional regulatory analysis, based on co-expressed gene clusters, during HUVEC senescence clarified hub genes as well as canonical and non-canonical responses, including E2F and SIRT2 signaling, which were down-regulated in lipid metabolism and chromosome organization processes. Besides changes in the expression of canonical splicing factor genes, novel splicing alterations such as AFE- and ALE-type splicing and microexons were identified. Our findings define a comprehensive transcriptome network, including gene expression and splicing, in the complex mechanisms of replicative endothelial senescence.

## Materials and methods

### Cell culture

HUVECs (Lonza, Basel, Switzerland) were cultured using an EBM-2 Bullet Kit (Lonza) at 37 °C in an atmosphere containing 5% CO_2_. Cells were seeded and subcultured 12–15 times at a confluence of 80–90% and density of 2–5 × 10^5^ cells in 100-mm diameter dishes until reaching senescence. Cells were maintained with medium changes once in 2–3 days. The PD rate was calculated using the following equation [45]:

$$PD=\frac{\text{ln}\left(No. of cells harvested\right)-\text{ln}\left(No. of cells seeded\right)}{\text{ln}2}$$

Then, HUVECs were subcultured at 37 °C in an atmosphere containing 5% CO_2_.

### SA-β-Gal staining

Cell fixation and SA-β-Gal staining were conducted using Cellular Senescence Assay Kit (CBA-230) according to the manufacturer’s instructions (Cell Biolabs, Inc., San Diego, CA, USA). Briefly, cells were fixed with Fixing Solution for 5 min at room temperature, washed with phosphate-buffered saline, and then stained with Cell Staining Working Solution overnight at 37 °C in the dark. SA-βgal–positive cells were identified using Image J (NIH, Bethesda, MD, USA). Four fields were examined per sample.

#### RNA-seq analysis

To comprehensively investigate transcriptome consequences during HUVEC senescence, two different HUVEC clones of HUVECs (C1 and C2) cultured for four different durations were analyzed by RNA-seq. The four timepoints selected for RNA-seq analysis included day 5 or 6 (youngest timepoint), day 18 or 20 (dividing phase), day 60 or 61 (when HUVECs stopped dividing; PD < 0.1 per day), and day 74 (oldest timepoint; 2 weeks after HUVECs stopped dividing). RNA-seq libraries were aligned to the hg19 (GRCh37) reference genome assembly using the Genomic Short-read Nucleotide Alignment Program (GSNAP) [46, 47]. Next, the mate-pair information of the aligned libraries was fixed, potential PCR duplicates were removed, and libraries were sorted using SAMtools [48]. Then, gene expression was quantified (as FPKM values) using Cufflinks [49]. Differential splicing was detected using MISO analysis [50] and VAST-TOOLS [31]. In MISO analysis, the following parameters were used for removing AS events: Bayes factor < 10, variation in percent spliced-in (|ΔPSI|) < 0.1, and zero reads supporting the inclusion or exclusion isoform or less than 10 reads supporting either of the event isoforms. Conjoined genes (readthrough transcripts) were identified using the deFuse software (omicX, France) [32], with the classifier modified by removing the est_breakseqs_percident and breakseqs_estislands_percident features since the inclusion of these classifier features can decrease the probability of gene fusion calls. Conjoined genes were identified by selecting deFuse gene fusion calls, where both participating genes were located on the same strand of the same chromosome, using the following parameters: deletion = “Y”; splice score = 4; exonboundaries = “Y”; probability ≥ 0.9.

The RNA-seq data of the neuronal differentiation in human iPSCs were obtained from the Gene Expression Omnibus database (GSE32625) [25]. The raw sequence data were aligned to the hg19 reference genome using GSNAP and analyzed using Cufflinks and MISO analysis (as described above).

### Co-expression network analysis

Gene expression response profiles were clustered using the WGCNA R software [26]. Soft-threshold selection was facilitated by calculating the scale-free network topology model fit *R*^2^ values for soft thresholds 1–30 using the pickSoftThreshold function. The final threshold value was manually selected, where the topology model fit was both relatively stable and high.

Genes with FPKM ≥ 1 in at least one sample of the C1 and C2 RNA-seq data sets were selected for clustering. Gene expression profiles were clustered using WGCNA R with the following parameters: networkType = “signed”; minModuleSize = 30. Genes in the RNA-seq data sets of both C1 and C2 clones were classified into 20 co-expression clusters using the following parameters: soft power threshold = 12; deepSplit = 0.

### Biological process enrichment analysis and enrichment map generation

To determine whether the expression of known senescence-related genes is altered during HUVEC senescence, enrichment analysis was conducted using senescence gene sets extracted from MSigDB, REACTOME, and HAGR databases, as well as genes of both HUVEC clones (C1 and C2) showing differential expression between the youngest timepoint (day 5) and oldest timepoint (day 74).

The Gene Ontology (GO) biological process term enrichment for a set of genes was conducted by generating functional interaction networks with the BiNGO Cytoscape plug-in [51] using genes in the resulting network. The GO terms were selected using the following thresholds: *P* = 1.0e^−7^; false discovery rate = 1.0e^−3^; overlap coefficient = 0.6. Enrichment maps were generated for the BiNGO biological process enrichment results using the EnrichmentMap Cytoscape plug-in [52].

### Transcriptional regulatory analysis

Transcriptional regulatory factors were predicted using upstream analysis with the IPA database (Qiagen, Qiagen Redwood City, CA, USA; www.qiagen.com/ingenuity) and enrichment analysis with the TFBS database. To conduct the TFBS enrichment analysis, the MSigDB compendium of transcription factor target gene sets was used. The enrichment significance was evaluated by hypergeometric statistics [20].

### Hub gene analysis

Genes with high intramodular connectivity (kWithin > 0.8) were defined as hub genes. The kWithin, which was calculated using WGCNA, represents the scaled degree of connectivity of the edge of the gene under the same module.

### RT-PCR

Total RNA was extracted using the RNeasy Plus Mini kit (Qiagen), and cDNA was synthesized using the SuperScript VILO cDNA Synthesis Kit (Life Technologies Corporation, CA, USA) or the PrimeScript RT reagent Kit (Takara Bio Inc., Japan), according to the manufacturers’ instructions. Real-time PCR was conducted using TaqMan MGB probe (Life Technologies), and the amount of FAM fluorescence was measured as a function of the PCR cycle number (C_T_) using the Applied Biosystems ViiA7 Real-Time PCR system (Life Technologies). To examine microexon alterations, primers were designed to contain alternative exons using Primer3 (http://bioinfo.ut.ee/primer3-0.4.0/). RT-PCR was conducted using AmpliTaq Gold (Life Technologies) using the following conditions: an initial denaturation step at 95 °C for 5 min, followed by 35 cycles at 94 °C for 30 s, 60 °C for 30 s, and 72 °C for 45 s, and a final elongation step at 72 °C for 7 min. The PCR products were separated by capillary electrophoresis, and signal intensity was measured using the LabChip GX Touch Nucleic Acid Analyzer (PerkinElmer, Inc., MA, USA). Hs01060665_g1 (Life Technologies) for β-actin, Hs00923894_m1 (Life Technologies) for CDKN2A, and the following primers and probes were used for amplification: ACACA39440 (probe): 5′-ATCGCCCGACCGCACACGTTGC-3′; ACACA39440-F: 5′-GCACGCCTGTCAGCCATC-3′; ACACA39440-R: 5′-TCCACTTCCAGAAAGACCTCAG-3′; ACACA35313 (probe): 5′-CACCACATCCTCTCATCATTGCGCCTCA-3′; ACACA35313-F: 5′-GGTGAAGAGGGTGCGTTTCA-3′; ACACA35313-R: 5′-CCCTCAAGATTGACATCAGAGTAGA-3′; PSAP-F: 5′-TTGCTATCCAGATGATGATGC-3′; PSAP-R: 5′-CCTCATCACAGAACCCAACC-3′; PRUNE2-F: 5′-GTCATCGAGCCCTACAGGAG-3′; PRUNE2-R: 5′-GCATTTAGACCGTCCCCATA-3′.

## Supplementary Information

**Additional file 1.**

**Additional file 2.**

**Additional file 3.**

**Additional file 4.**

## Data Availability

RNA-seq raw sequencing data are available at GEO (GEO series accession number: GSE163251) and all data generated or analyzed during this study are included in this published article and its supplementary information files.

## Abbreviations

SA-β-Gal: Senescence-associated β-galactosidase
AS: Alternative splicing
HUVEC: Human umbilical vein endothelial cell
AFE: Alternative first exon
ALE: Alternative last exon
MSigDB: Molecular Signatures Database
HAGR: Human Ageing Genomic Resources
DEG: Differentially expressed gene
ASG: Alternatively spliced gene
FPKM: Fragments per kilobase of exon model per million reads mapped
iPSC: Induced pluripotent stem cell
WGCNA: Weighted Correlation Network Analysis
IPA: Ingenuity pathway analysis
TFBS: Transcription factor binding site
A3SS: Alternative 3ʹ splice site
A5SS: Alternative 5ʹ splice site
MXE: Mutually exclusive exon
RI: Retained intron
SE: Skipped exon
RT-PCR: Reverse transcription PCR
GSNAP: Genomic Short-read Nucleotide Alignment Program
GO: Gene Ontology
