# Supplementary material for: Gene regulatory network analysis defines transcriptome landscape with alternative splicing of human umbilical vein endothelial cells during replicative senescence
Source: BMC Genomics. 2021 Dec 2;22:869. doi: 10.1186/s12864-021-08185-x (PMC8641155; doi:10.1186/s12864-021-08185-x)
Supplement: Supplementary file 1 — Additional file 1. [file 12864_2021_8185_MOESM1_ESM.pdf]

Gene regulatory network analysis defines transcriptome landscape with alternative splicing of human umbilical vein endothelial cells during replicative senescence

Momoko Ohori[1,6]*, Yusuke Nakayama[1,4,6], Mari Shimizu-Ogasawara[1], Hiroyoshi Toyoshiba[1,5], Atsushi Nakanishi[1], Samuel Aparicio[2,3], Shinsuke Araki[1]*

[1]Research, Takeda Pharmaceutical Company Limited, 26-1, Muraoka-Higashi 2-chome, Fujisawa, Kanagawa, Japan

[2]Molecular Oncology, BC Cancer Agency, 675 W10th Avenue, Vancouver, BC, V5Z 1L3, Canada

[3]Department of Pathology and Laboratory Medicine, University of British Columbia, Vancouver, BC, V6T 2B5, Canada

[4]Present address: Discovery Technology Research Laboratories, Tsukuba Research Institute, Ono Pharmaceutical Co., Ltd., 17-2 Wadai,
Tsukuba, Ibaraki 300-4247, Japan

[5]Present address: Research Department, FRONTEO Healthcare Inc., 2-12-23 Konan, Minato-ku, Tokyo 108-0075, Japan

[6]These authors contributed equally

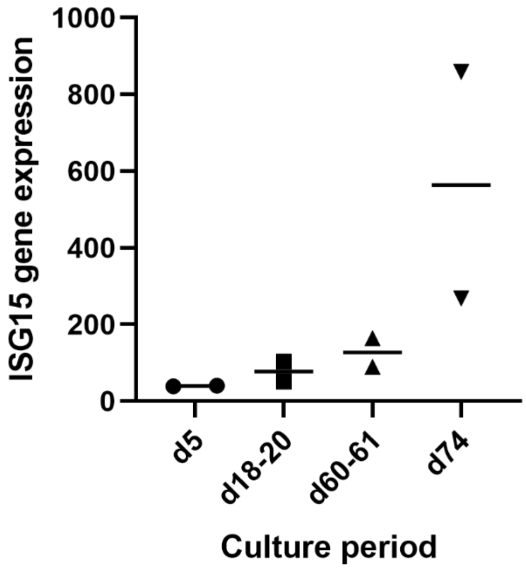

Fig. S1. Gene expression of ISG15 from RNA-seq data during HUVEC senescence in C1 and C2 individual clones of each culture period (e.g., day 5, 18, 60, 74).

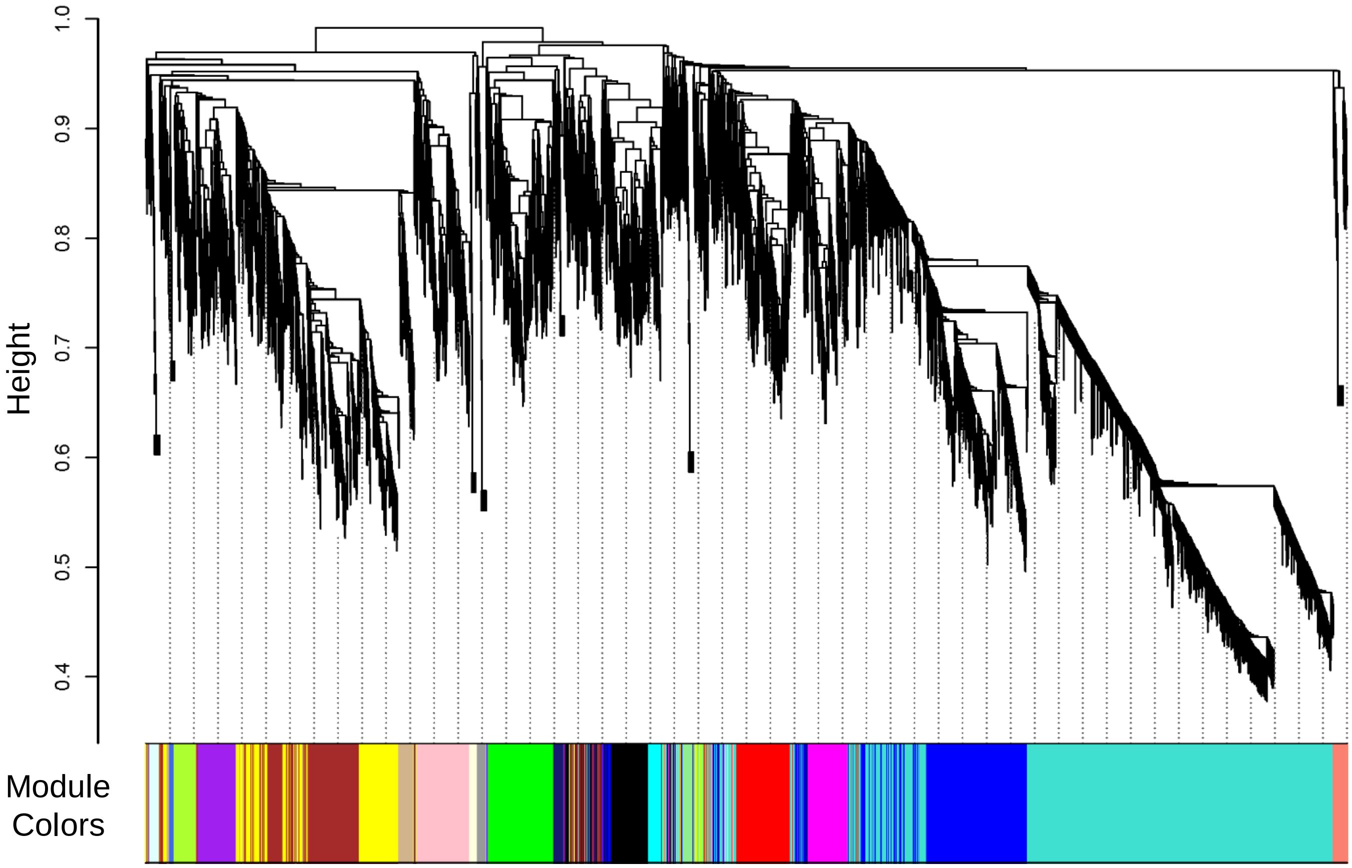

Fig. S2. WGCNA cluster dendrogram showing 20 modules of co-expressed genes. Colored bars consistent with the module name (color) for the clustered genes.

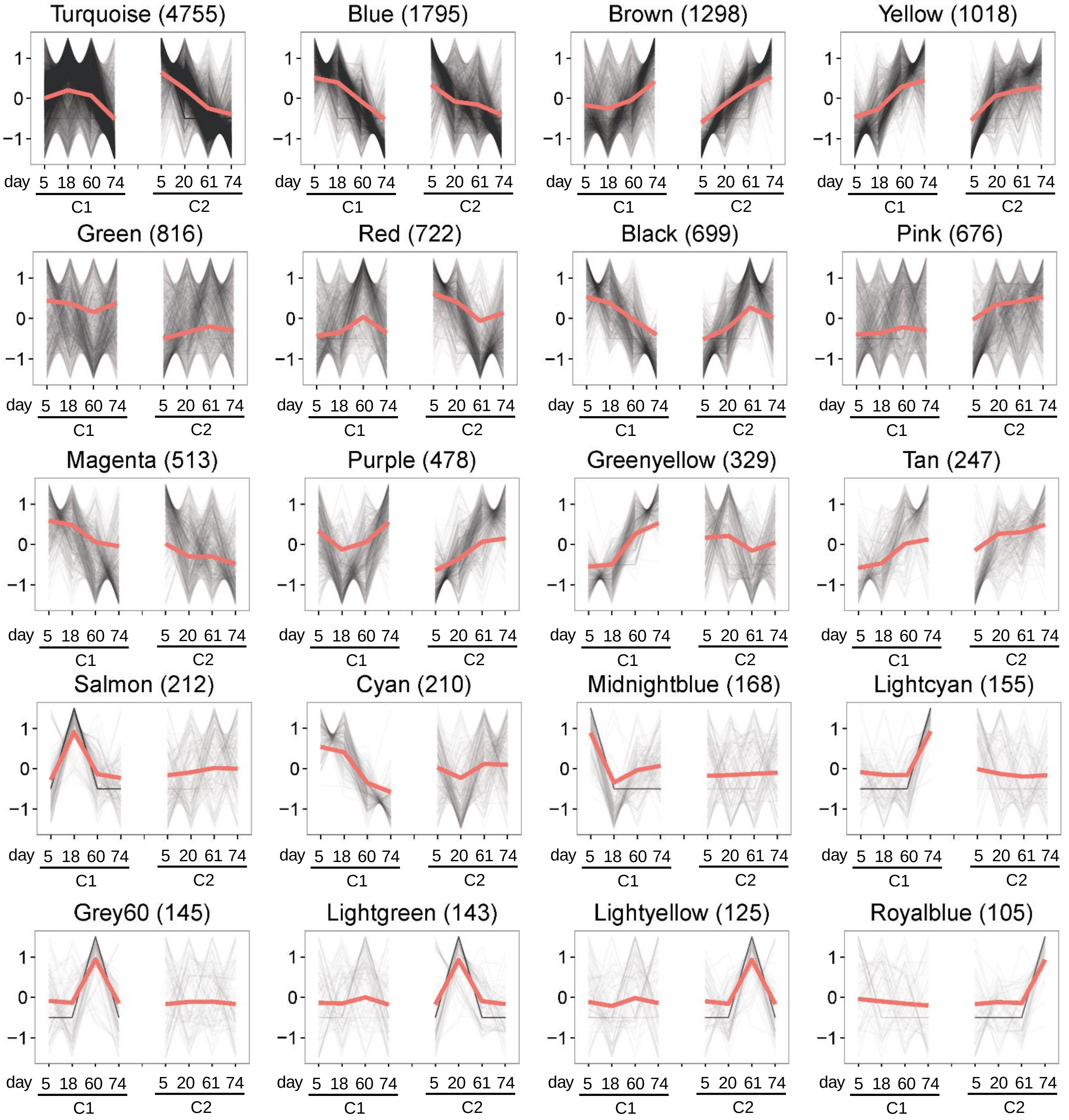

Fig. S3. Expression patterns for genes of co-expressed clusters from WGCNA analysis based on FPKM values in HUVECs with different culture periods. The Y axis indicates the standardized FPKM value. Black lines represent the value of individual genes. The red line indicates the value of eigengenes for each module. The number of genes in each module is shown in parentheses in the module label. WGCNA, weighted correlation network analysis; FPKM, fragments per kilobase of exon model per million reads mapped; HUVECs, human umbilical vein endothelial cells.

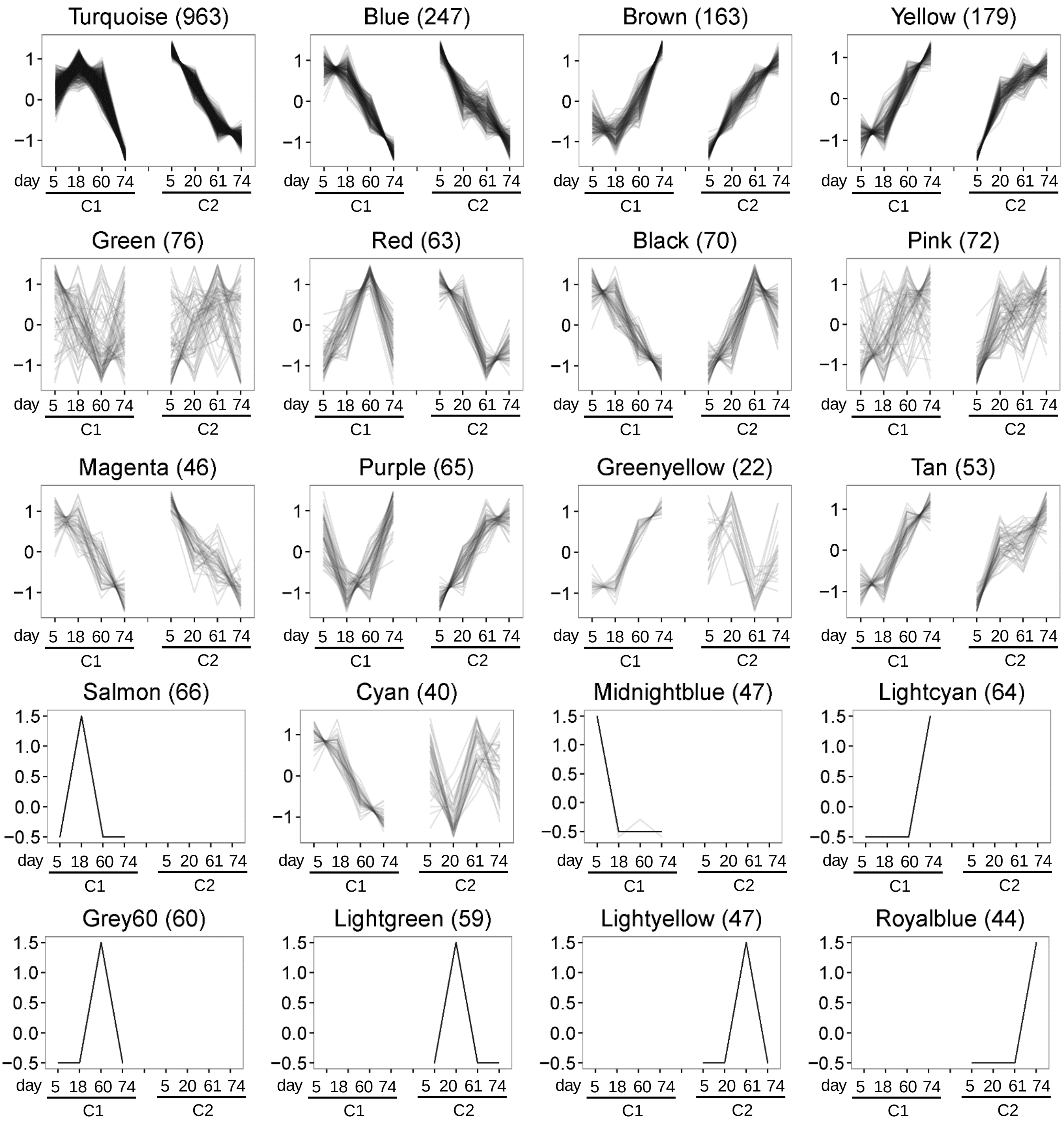

Fig. S4. Expression pattern of each module for hub-genes. Heat map indicating P-values of enrichment analysis for four dominant modules (turquoise, blue, brown, and yellow) against known senescence gene sets (Table S2).

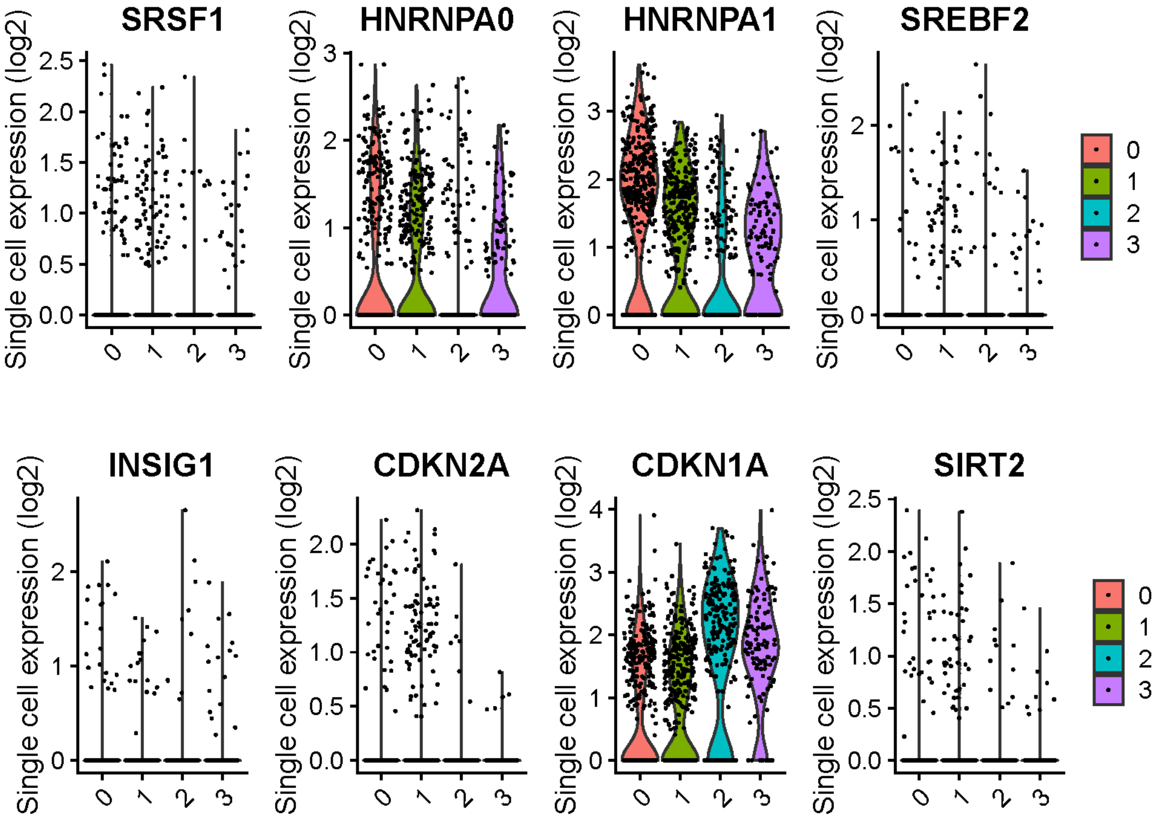

Fig. S5. Single cell level of gene expression of senescence core for each cluster (0-3).

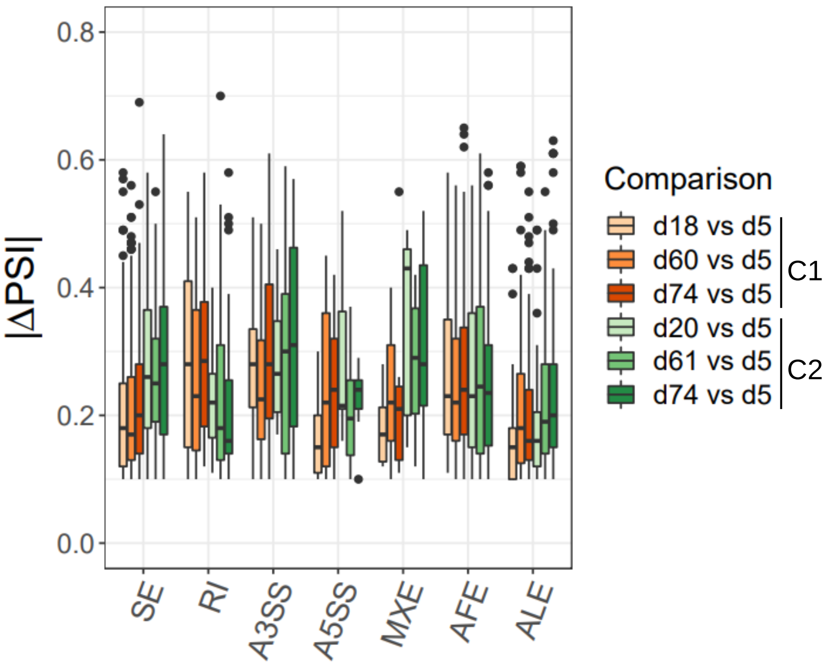

Fig. S6. ΔPSI value of splicing event type in HUVECs in each culture period as determined by MISO analysis.

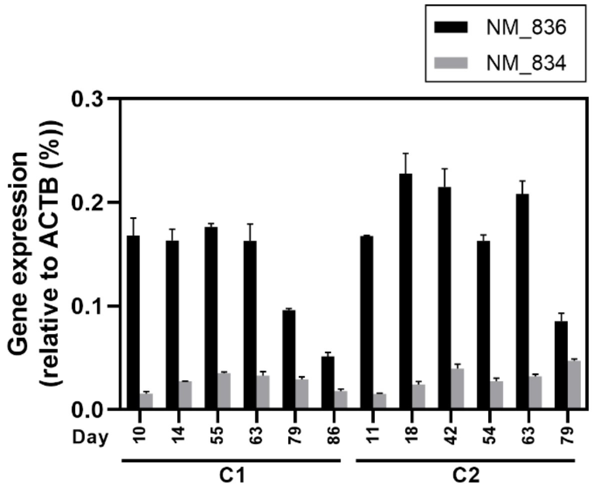

Fig. S7. Analysis of ACACA gene expression by qPCR for C1 and C2 clones. The Y-axis indicates relative gene expression for NM_198836 [NM_836] or NM_198834 [NM_834]. *ACTB* mRNA expression was evaluated as an internal control. The X-axis indicates culture days. Data represent mean ± SD of two independent experiments.
